# Chromosomal Rearrangements and Altered Nuclear Organization: Recent Mechanistic Models in Cancer

**DOI:** 10.3390/cancers13225860

**Published:** 2021-11-22

**Authors:** Concetta Federico, Francesca Bruno, Denise Ragusa, Craig S. Clements, Desiree Brancato, Marianne P. Henry, Joanna M. Bridger, Sabrina Tosi, Salvatore Saccone

**Affiliations:** 1 Department of Biological, Geological and Environmental Sciences, University of Catania, Via Androne 81, 95124 Catania, Italy; federico@unict.it (C.F.); francesca.bruno@unict.it (F.B.); desiree.brancato@phd.unict.it (D.B.); 2 Leukaemia and Chromosome Research, Centre for Genome Engineering and Maintenance, College of Health, Medicine and Life Sciences, Brunel University London, Kingstone Lane, Uxbridge UB8 3PH, UK; denise.ragusa2@brunel.ac.uk (D.R.); sabrina.tosi@brunel.ac.uk (S.T.); 3 Laboratory of Nuclear and Genomic Health, Centre for Genome Engineering and Maintenance, College of Health, Medicine and Life Sciences, Brunel University London, Kingstone Lane, Uxbridge UB8 3PH, UK; craig.clements@cambridge.org (C.S.C.); marianne.henry17@gmail.com (M.P.H.); Joanna.Bridger@brunel.ac.uk (J.M.B.)

**Keywords:** cancer, genome organization, chromosomal rearrangements, topologically associated domains, replication timing

## Abstract

**Simple Summary:**

New methodologies and technologies developed in the last few decades have highlighted the precise spatial organization of the genome into the cell nucleus, with chromatin architecture playing a central role in controlling several genome functions. Genes are expressed in a well-defined way and at a well-defined time during cell differentiation, and alterations in genome organization can lead to genetic diseases, such as cancers. Here we review how the genome is organized in the cell nucleus and the evidence of genome misorganization leading to cancer diseases.

**Abstract:**

The last decade has seen significant progress in understanding how the genome is organized spatially within interphase nuclei. Recent analyses have confirmed earlier molecular cytogenetic studies on chromosome positioning within interphase nuclei and provided new information about the topologically associated domains (TADs). Examining the nuances of how genomes are organized within interphase nuclei will provide information fundamental to understanding gene regulation and expression in health and disease. Indeed, the radial spatial positioning of individual gene loci within nuclei has been associated with up- and down-regulation of specific genes, and disruption of normal genome organization within nuclei will result in compromised cellular health. In cancer cells, where reorganization of the nuclear architecture may occur in the presence of chromosomal rearrangements such as translocations, inversions, or deletions, gene repositioning can change their expression. To date, very few studies have focused on radial gene positioning and the correlation to gene expression in cancers. Further investigations would improve our understanding of the biological mechanisms at the basis of cancer and, in particular, in leukemia initiation and progression, especially in those cases where the molecular consequences of chromosomal rearrangements are still unclear. In this review, we summarize the main milestones in the field of genome organization in the nucleus and the alterations to this organization that can lead to cancer diseases.

## 1. Introduction

In all organisms, including bacteria [1], the genome is organized within a “nuclear space” to facilitate the genes’ switching on and off, transcriptional regulation, and irreversible silencing of genes. In eukaryote cells, this requires a highly organized nuclear structure, with chromatin architecture playing a central role in maintaining healthy cells. A further layer of genome organization in the cell nucleus, alongside the DNA sequence itself and a defined chromatin structure, is the spatial positioning of defined regions, sometimes also known as spatial epigenetics [2], whereby individual chromosomes, single chromosomal bands, clusters of genes and individual gene loci reside in nonrandom locations within cell nuclei. Alterations to this genomic organization in the nucleus could lead to genetic diseases, including cancer, as will be disclosed in the following sections.

In the last two decades, chromosomes have been intensely studied inside the cell nuclei to understand if they occupy specific positions to correctly drive the expression of genes they contain. Radial chromosome positioning is evident in all animal cells, as demonstrated in human [3,4,5,6], mouse [7,8], primates [9,10], canine species [11], pigs [12,13], birds [5,11,14,15], reptiles, and amphibians [16]. Overall, from these studies, it appeared that the spatial organization of these animals’ genomes placed less transcriptionally active regions of the genome close to the nuclear periphery and more transcriptionally active regions in the inner part of the nuclei [5,6,17,18,19,20,21]. Of course, it follows that genes housed upon individual chromosomes would also be radially and nonrandomly located in cell nuclei, and such positioning for individual gene loci has indeed been observed numerous times for different organisms [2], for example, in humans [6,22,23,24,25,26,27], mouse [23], pig [28,29], and snail [30]. Thus, the chromosomal organization in the cell nucleus and the chromatin architecture are evolutionarily highly conserved in mammals and birds to maintain the correct functioning of the genome. In humans, this well-controlled genome organization is sometimes subject to several types of alterations, following chromosomal rearrangements, that can lead to the modification of normal gene distribution in the nucleus (see next sections). These variations in chromatin organization can be found in several genetic diseases, such as cancer [2].

Here, we review the recent advances in knowledge about gene and genome organization in the cell nucleus. We focus on the organization of genes in the nuclear chromosome territories and variation in the chromatin structure as a consequence of chromosomal rearrangements leading to repositioning in the nucleus of specific genes. This repositioning can lead to an ectopic alteration in gene expression and replication timing of the involved genomic sequences, two features related to cancer diseases.

## 2. The Chromosome Organization in the Cell Nuclei

Using DNA probes to “paint” individual chromosomes with the fluorescence in situ hybridization (FISH) technique, it has been possible to visualize whole chromosome territories in the interphase nuclei allowing precise analyses to understand the organization of each chromosome in the nucleus [31]. Indeed, it was determined that individual chromosomes occupy specific radial positions according to their average gene density in proliferating cells [3]. This was demonstrated, for the first time, for two small human chromosomes, chromosomes 18 and 19, endowed with opposing properties taking into consideration the GC level of the genomic sequence and gene density. The authors found that the chromosome territory of the gene-poor/GC-poor chromosome 18 was located at the nuclear periphery, whereas the territory of the gene-rich/GC-rich chromosome 19 was located more internally in the nucleus [3,17]. The spatial organization in the cell nuclei of these two chromosomes has also been observed in other organisms such as mice [32] and primates [9], where the syntenic chromosomes show similar nuclear distribution.

**Figure 1:**
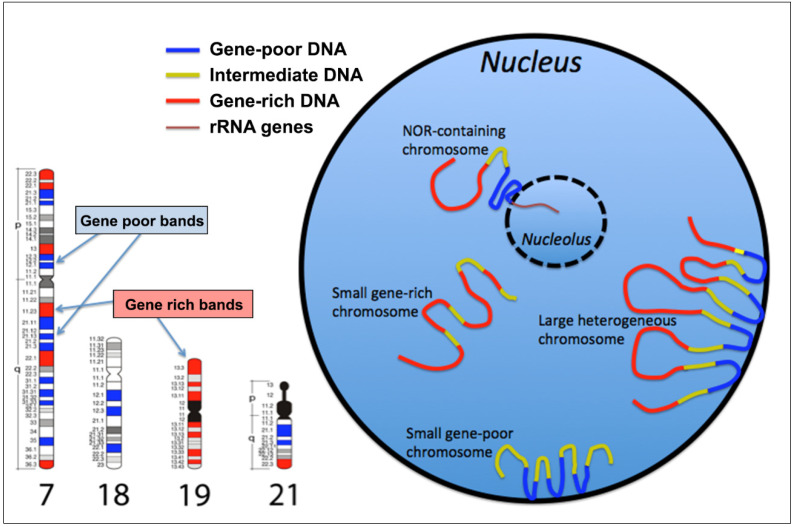
Chromosome organization in the cell nucleus. The left panel displays ideograms of four representative human chromosomes. Red and blue bands indicate the gene/GC-richest and the gene/GC-poorest bands, respectively. The other bands are endowed with intermediate properties. The right panel displays a schematic representation of the chromosomal band sequence distribution through interphase nuclei according to their genomic properties with respect to gene/GC content. Small chromosomes can be located at the nuclear edge or in the interior, with gene-poor chromosomes being located at the nuclear edge and gene-rich in the interior, in proliferating cells. Large chromosomes, generally heterogeneous in their genomic properties (such as the human chromosome 7), assume a “zig-zag” conformation and are positioned with the GC-richest bands exposed towards the interior of nuclei. Nucleolus organizer regions (NOR) containing chromosomes (such as the human chromosome 21) have associations with internal nuclear structure with the GC-poorest bands located close to the nucleoli.

Chromosomes 18 and 19 occupy different intranuclear positions (the former more peripheral and the latter more internal) because they are small and relatively homogeneous in their nucleotide composition and gene density, as previously shown [33,34,35,36]. Thus, these two chromosomes can occupy different nuclear locations due to their small size and different genomic features (Figure 1). On the other hand, larger chromosomes, being highly heterogeneous in terms of nucleotide composition and gene distribution, cannot occupy a specific position close to the nuclear periphery or more internally in the nucleus [6]. In this case, because the genomic regions with opposite properties are not contiguous but distant from each other, large and heterogeneous chromosomes can organize their different chromosomal regions using a “zig-zag” distribution to position all the GC-poorest DNA at the nuclear periphery and all the GC-rich DNA toward the inner part of the nucleus [6]. Genomic DNA with intermediate properties connects the above regions (Figure 1). Confirmation of this chromosomal organization in the nucleus was also obtained by localization in the cell nucleus of the compositionally fractionated DNA used as a probe in FISH experiments: the GC-rich DNA was located in the inner part of the nucleus. At the same time, the GC-poor was observed only in the nuclear periphery. This means that all GC-rich chromosomal bands reside in the nuclear interior, even if they belong to different chromosomes and even if they occupy different positions along each chromosome. 

Conversely, all the GC-poor bands, along with the centromeric sequences, are located at the nuclear periphery or close to the nucleolar periphery [3]. Indeed, in this latter case, chromosomes containing the rRNA gene clusters (Nucleolus organizer regions (NOR)), namely chromosomes 13, 14, 15, 21, and 22, reside close to the nucleolus, thus in these cases, the entire chromosomes are located in the inner part of the nucleus. However, their gene/GC-poorest bands maintain the same functional position in the peripheral part of the nucleolus, composed primarily by the centromeric and pericentromeric heterochromatin of the NOR-containing chromosomes. The rDNA clusters located in the short arm of these chromosomes are organized in a rosette mode, thus determining the nucleolus formation.

These results demonstrate that chromosome territories are endowed with a GC-level gradient increasing from the periphery to the inner nuclear compartment (Figure 1). This result was confirmed by further studies [37], showing the active gene sequences located in chromatin loops stretching into the nuclear interior and the gene-poor sequences located at the nuclear periphery.

More recent techniques were developed to study the organization of the genome in 3D within the cell nucleus. These are derived from the chromosome conformation capture (3C) method [19], namely the variants 4C (chromosome conformation capture-on-chip) [38], 5C (chromosome conformation capture carbon copy) [39], and Hi-C (high-throughput chromosome conformation capture) [40]. This latter variant, Hi-C, has greatly enhanced our knowledge of the nuclear chromatin architecture. Indeed, these methodologies have revealed the closeness of individual genomic sequences to other sequences by cross-linking of chromatin regions that are joined or very close to one another. In this way, intra- and inter-chromosomal contacts can be determined, and thus a 3D model of chromosome and gene interactions was developed. Hi-C has given us the most extensive view of the 3D organization of the genome as all interactions between chromosomal regions are sequenced and considered when the computer-generated map of the genome is constructed [41].

The analysis of the human genome’s spatial organization through Hi-C confirmed what was initially found by FISH, namely that gene-rich chromosomal regions are spatially linked, as demonstrated by the high number of contacts observed [40]. Interestingly, gene-poor chromosomes 13 and 18 make very few contacts with other gene-rich chromosomes such as 17, 19, and 22, confirming that chromosomes, and more precisely chromosomal bands, share similar or different positions based on their gene density [3,5].

The Hi-C data continued to enhance our knowledge on chromatin organization in the cell nuclei, revealing the presence of Topologically Associated Domains (TADs), a structural organization of the chromatin endowed by a loop structure where the zinc finger CCCTC-binding factor (CTCF) sites play an essential role [42,43]. CTCF sites are critical in TAD organization, with these sites being located at the base of the TAD loops, aiding in the insulation of the sequence present inside each loop. Thus, a TAD loop could be considered not only a structural but also a functional region of the genome isolated from the other neighbor TADs [44], and dysfunction of TADs is involved in human diseases [45] and leukemogenesis [46].

These biochemical–molecular approaches have disclosed the presence in interphase of two genomic compartments A and B [40], organized into TADs of various sizes, with the former compartment located more internally in the nucleus and the latter more towards the nuclear periphery and surrounding nucleoli [47,48], where heterochromatin is located. These studies clearly showed the correspondence of compartment A and B with the GC-richest and GC-poorest chromosomal band DNA, respectively [44]. Thus, information obtained with different methodologies (biochemical and cytogenetic), joining data from the high-resolution molecular Hi-C method (at Kb level resolution) to that involving molecular cytogenetic techniques (with a lower level of resolution), demonstrated a unified model of the organization of chromosomal DNA in the interphase nucleus. This has highlighted the correspondence of the GC-rich isochores (and the GC-rich chromosomal bands) with the TADs located more internally in the cell nucleus. On the other hand, the GC-poorest isochores, corresponding to the GC-poor chromosomal bands, correspond to the genomic regions with the highest number of TADs specifically located close to the nuclear envelope and thus also defined as Lamina Associated Domains (LADs) [44,49].

## 3. Genome Organization inside the Chromosome Territories

Since genes are part of chromosomes, it also matters where those genes sit in relation to the body of their home interphase chromosome territory, i.e., intrachromosomal organization. Indeed, gene loci can be located deep within chromosome territories, more towards the surface of the chromosome territories, or even at some distance from the main body of the chromosome territory, out on a chromatin loop. There is evidence that even within interphase chromosomes, there is the spatial organization of gene-rich and gene-poor areas, which has been revealed by FISH, with inactive genes more likely to be located in the interior of chromosome territories [6,23,50,51,52,53,54]. When chromosomes are located at the nuclear periphery, their active genes are generally pointed towards the nuclear interior and not the nuclear envelope side [6,37].

In highlighting the importance of intrachromosomal gene location, it is worth pointing out the case of the over-expressed genes *MYC* and *CCND1* in cancer cells located at the edges of their chromosome territories. The same genes, when silenced, were located deep within the interior of the chromosome territories. Experimentally silencing the over-expressed genes leads to alterations in the genes’ locations, which become more internal in the chromosomal territories [55]. This implies some flexibility and fluidity within the chromosomal territory in gene distribution according to their transcriptional activity. How this would naturally occur would need to be further investigated, but it would probably be controlled by chromatin remodeling via histone modification [56]. Moreover, genes located inside the chromosome territories are less likely to form interchromosomal translocations but are more prone to forming intrachromosomal exchanges [57].

The arrangement of genes within a chromosome territory would permit the active regions of the genome to be closer and exposed to the components, machinery, and structures required for transcription and processing [58]. We know that active transcription can occur at the surfaces and around channels that inveigle their way into chromosomes [59]. However, some gene loci are located away from the main body of their chromosome territory out on loops to be transcribed at a distance from the chromosome [6,53,60,61], at transcription factories, or splicing speckles [28,62], becoming colocalized with other genes [21,63]. This positioning of chromatin loops belonging to different chromosomes in the same nuclear compartments can determine chromosomal rearrangements such as translocations.

## 4. Nuclear Architecture, Replication Timing, and Cancer Diseases

Replication timing refers to the ordered succession in which the genome is duplicated during the S phase of the cell cycle. The replication does not proceed from one end of a chromosome to the other, but it starts at different genomic sites and proceeds in a well-defined order based on the genomic properties of the different chromatin regions [64,65,66]. Genomic regions with similar features, also belonging to different chromosomes, are replicated simultaneously during the S phase [67,68], starting in the inner part of the nucleus and ending at the nuclear periphery, highlighting a clear relationship between replication timing and the three-dimensional organization of the chromatin according to the GC-level and to the gene density [69]. Indeed, a higher gene density correlates to earlier replication during the S phase, while low gene density undergoes late replication [70,71], with heterochromatin and centromeric sequences replicated in later stages of the S phase [72].

**Figure 2:**
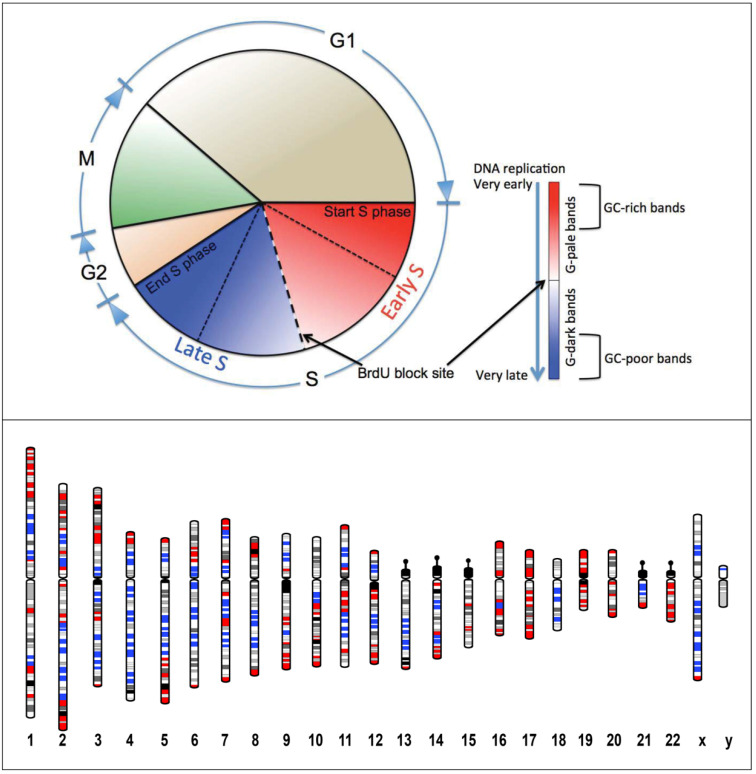
Replication timing of the human chromosomal bands. Upper panel. Scheme of the cell cycle with the S phase and its different temporal sections highlighted. S phase is the period when the DNA is replicated and could be divided into two parts taking into consideration the block of the cell cycle that happens when the cells grow in a medium with a high concentration of bromodeoxyuridine (BrdU). In this way, the early S phase corresponds to the period in which DNA from the GC-rich pale G-bands is replicated, and the late S-phase is the period in which the GC-poor DNA from the dark G-bands is replicated. Moreover, the gene-richest bands are replicated at the beginning of the S phase (very early replicated DNA), and the gene-poorest bands at the end (very late replicated DNA). The remaining bands, endowed with an intermediate gene density, are replicated in the middle part of the S phase, first the G-pale bands and then the G-dark bands. Bottom panel. Human chromosome ideograms with G-bands showing the GC-richest and the GC-poorest bands [35,36] highlighted by red and blue colors, respectively. Note that the GC-richest bands (red colored) and the GC-poorest bands (blue colored), namely the two band sub-sets replicated in two very different subperiod of the S phase, are generally not adjacent. The other bands, with intermediate compositional properties, are replicated in the central part of the S phase.

The Association of replication timing and chromosomal bands has been investigated by incorporating 5-bromodeoxyuridine (BrdU) into newly replicated DNA. DNA from different chromosomal band types is replicated at different times during the S phase of the cell cycle, with human chromosomal bands classified into 18 replication groups, according to their replication timing, with the first group replicated at the beginning of the S phase and the last at the end [73]. Incidentally, the chromosomal bands endowed with the highest and the lowest GC levels largely corresponded to the chromosomal bands replicated in the first three and the last three replication groups. In other words, the GC-richest bands corresponding to the gene-richest bands are replicated at the beginning of the S phase of the cell cycle (Figure 2) [74]. Thus, the genomic compartment located towards the inner part of the nucleus presents an open chromatin structure, containing most of the active genes endowed with a high GC level, and is mainly replicated at the onset of the S phase [74,75,76,77]. The opposite properties were observed in the genomic compartment located at the nuclear periphery.

Alterations in replication timing patterns are observed in several diseases, including constitutional syndromes and cancer [78]. Biallelically expressed genes display a synchronous pattern of replication timing, whereas monoallelically expressed genes, such as imprinted genes and genes on the X chromosome, show an asynchronous pattern of replication [79,80,81,82]. Abnormal replication timing was also described in microdeletion syndromes, namely constitutional disorders characterized by the monoallelic deletion of several genes in a certain genomic fragment of approximately 1-5Mb in size. A delayed replication timing of the one remaining copy of the *HIRA*/*Tuple* gene on 22q11.21, related to the DiGeorge/velocardiofacial syndrome, was observed [24]. Thus, changes in the replication timing can be considered a sign of disruption to normal genome organization that can impact the integrity of the nuclear architecture and correct gene expression.

Cancer-associated genes such as *RB1*, *AML1*, and *CMYC*, which display synchrony in allelic replication in normal cells, have shown earlier replication timing and asynchronous allelic replication in neurofibromatosis type 1 (NF1), a cancer-predisposing syndrome [83]. Similarly, loss of synchrony was also described in patients with nonalcoholic fatty liver disease and cryptogenic cirrhosis, which are premalignant conditions of liver cancer [84]. These findings suggest that replication timing abnormalities could represent initiating events in carcinogenesis. Replication asynchrony has been documented in various types of cancers, such as lymphoma, breast cancer, prostate, and colorectal cancer, and other hematologic malignancies [85,86,87,88,89], and abnormal replication timing has been proposed as a preceding or predisposing event for chromosomal translocations in leukemia. Ryba et al. [90] showed that acute lymphoblastic leukemia patients with no translocations presented changes in replication timing in regions that are prone to that type of lesion.

Replication timing patterns could potentially be employed as a diagnostic tool to precisely classify and predict diseases, but further studies are needed to establish precise protocols. Given the multifactorial nature of cancer, the replication timing could contribute to the development of the disease, although it is probably not the only initiator. Fritz et al. [91] reported an altered replication timing of cancer-related genes in malignant cells, whereas this was not the case in healthy and precancerous cells. This observation led to the conclusion that other mechanisms and factors, mainly epigenetic, probably contribute to the transformation process. It is also possible that alterations in replication timing result from the epigenetic changes and the alterations in the chromatin structure following chromosomal rearrangements.

## 5. Chromosomal Rearrangements and Nuclear Gene Repositioning in Cancers

Structural chromosomal abnormalities are a hallmark of several cancers, and chromosomal rearrangements involve significant alterations and exchanges of genomic material [92]. The molecular consequences of chromosomal rearrangements that affect genes located at the breakpoints have been extensively studied. However, the consequences and the impact of the alterations that do not directly disrupt gene(s) structure (i.e., those cases in which the breakpoints are located in the intergenic regions) but determine a spatial reorganization of the genome in the nucleus remain under-explored. Indeed, balanced rearrangements such as translocations and inversions can lead to a change in the regulatory context of the genes affected or to the formation of oncogenic chimeric genes, but in several cases, no phenotypic effect is immediately apparent.

However, the consequences of many recurrent chromosomal abnormalities remain unknown. Several studies have explored the link between chromosomal abnormalities and their effect on gene expression [reviewed in [93]]. Due to the precise nature of chromosome positioning within the nucleus, according to the GC-level of the genomic regions composing each band, the gross changes created by chromosomal rearrangements lead to major spatial disorders that go beyond local and in-cis effects. Chromosomal anomalies determine a change in the nuclear location of the chromosomal bands located around the breakpoints, and this spatial repositioning can cause changes in the accessibility status of the chromatin involved in the rearrangement, with repercussions on the transcriptional regulation of genomic loci not directly altered by the rearrangements [94].

**Figure 3:**
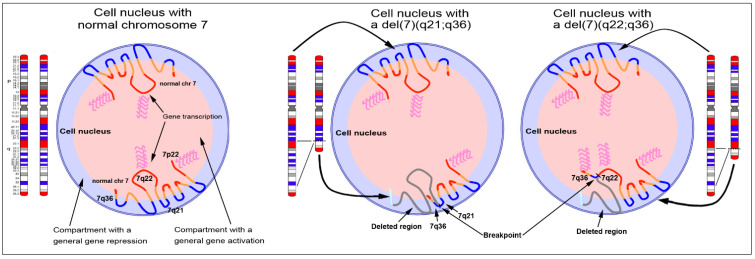
Effect of chromosomal rearrangements on gene expression. The image shows three nuclei with the zig-zag organization of the two chromosomes 7. Ideograms of the two homologous chromosomes 7 show the GC-richest (red) and the GC-poorest (blue) bands. The image shows three drawings of nuclei with two normal copies of chromosome 7 (**left**), one chromosome 7 carrying an interstitial deletion between 7q21 and 7q36 (**middle**), and one chromosome 7 with an interstitial deletion between 7q22 and 7q36 (**right**). Based on the new environmental conditions in which the terminal bands of chromosome 7 will be found after the deletion, activation of the genes involved in the rearrangement may occur, as observed for the *MNX1* allele in patients with hematological disorders. The nucleus on the right shows the case of gene activation by repositioning the 7q36 band in a transcriptionally active compartment (close to the GC-rich 7q22 band), as opposed to what is presented in the nucleus in the center, where the deletion involving the GC-poor 7q21 band keeps the 7q36 band in the same transcriptionally inactive compartment. Modified from [97].

Due to a chromosomal rearrangement (such as translocation, inversion, or deletion), genes located close to the breakpoints might alter their position in the nucleus, and this occurs when two chromosomal bands characterized by a very different GC-level, and normally located in different nuclear compartments, find themselves close together in the nucleus, with one of them located in a noncanonical nuclear compartment. Indeed, this is usually the case when two chromosomal bands endowed with opposing features (GC-rich/gene-rich vs. GC-poor/gene-poor) are involved in the rearrangement resulting in close contiguity of sequences normally positioned very far from each other. Depending on the cell type affected by the rearrangement, this may imply that a transcriptionally inactive gene could be ectopically activated by being positioned in a transcriptionally active nuclear compartment. Conversely, a gene could be inactivated if it is repositioned from the internal part of the nucleus to the nuclear periphery. In the case rearrangement happens between two compositionally similar genomic regions, the affected chromosomal bands retain the same radial position in the nucleus (Figure 3). This type of chromosomal rearrangements, and the functional consequences related to the aberrant nuclear location, was studied on both constitutional syndromes and cancer to focus on the relationship between the nuclear location of the derivative chromosomes, the gene expression, and the general context of the reorganized nuclear architecture [95,96].

On the Ewing sarcoma cells carrying a cancer-related translocation t(11;22), it has been noted that the fusion genes *EWSR1*/*FLI1* and *ABL1*/*BCR* were observed in an intermediate nuclear position when compared to the wild type *EWSR1* and *FLI1* genes and that this location seems to depend to the chimeric chromosomes obtained by the translocation [98]. However, it appears that there is no change in nuclear positioning if the genes affected by the translocation initially had a similar location. This is the case of *BCR* and *ABL* and the derivatives resulting after the formation of the Philadelphia chromosome [99]. Some authors analyzing wild type *MLL* gene and five of its translocation partners showed that the resulting fusion genes changed their nuclear location according to the gene density of the 2 Mb window of the regions involved [100]. Meaburn and Misteli [101] have demonstrated a change in the radial nuclear location of cancer genes in early tumorigenesis. However, interestingly, this was not associated with chromosomal rearrangements affecting the genes studied, and the change in nuclear position was not accompanied by an altered gene expression. These observations suggest that the repositioning of genes is prior to the change in gene expression, suggesting that a nuclear position effect is responsible for the perturbed transcription. A murine experimental model using *HOX* genes has provided evidence that nuclear reorganization does not depend on gene expression but can operate upstream to it [102].

Another interesting case concerns the t(7;12) translocation frequently observed on acute myeloid leukemia cells of pediatric patients, where the overexpression of the *MNX1* gene (previously called *HLXB9*) was related to its altered radial nuclear position as a consequence of the translocation of the 7q36 band (containing the *MNX1* gene) on the telomeric end of the chromosome 12 [25]. This translocation repositioned the *MNX1* gene more internally in the nucleus than the wild type allele. The ectopic activation and overexpression of *MNX1* were also described in relation to other chromosomal rearrangements observed on cell lines GDM1 [103] and K562 [27], as well as deletions observed in patients with hematological disorders [97]. In this latter case, the activated *MNX1* gene was related to the type of breakpoints in the deleted chromosome 7, namely when the deletion joined the region with the *MNX1* allele to a chromosomal band with high GC-level, thus finding a transcriptionally active nuclear compartment [97] (Figure 3).

The chromosomal organization in the cell nuclei with the t(11/22) translocation, further to the alteration in gene expression related to the altered spatial organization of the genes located close to the der (11) breakpoint, highlighted a different position in the nuclei and an altered gene expression profile also for genes located on other chromosomes not directly involved in the translocation [96]. This indicates that a chromosome rearrangement influences nuclear chromatin organization not only of the genomic regions surrounding the breakpoints or the chromosomes directly involved in the rearrangement but also the chromatin architecture corresponding to the nuclear territories of other chromosomes [59,100,104]. This appears to depend on long-range interactions between chromosomes in the cell nuclei, although translocations between neighboring chromosomes occur at a higher frequency, as observed by comparing the nuclear position of some loci in human lymphoma B-cells and the normal B-cells [104].

## 6. Conclusions

Cancer is a genetic disease related to the altered expression of genes generally involved in the cell cycle progression. A growing amount of evidence has shown that gene expression alteration observed in cancer cells could be related to a different position of genes in the cell nuclei and consequently to a chromosomal rearrangement. The nuclear position effect reflects the chromatin organization in the cell nuclei. Many different experimental procedures have demonstrated the presence of two very different nuclear compartments, one endowed with a high GC level, a high gene density, a very early replication during the S-phase of the cell cycle, and a position in the inner part of the nucleus. Another compartment, generally located more peripherally in the nucleus, is endowed with opposite properties. Rearrangements between loci belonging to these two different compartments influence the repositioning of genes in a compartment with different environmental properties, determining the ectopic activation or inactivation of the relocated genes. To date, only a few studies have focused on the nuclear positioning of genes in cancer, and further investigations should be carried out to better understand the real impact of this mechanism in the oncogenic process initiation and progression.

## References

[1] Rocha E.P.C. (2008). The Organization of the Bacterial Genome. Annu. Rev. Genet..

[2] Bridger J.M., Arican-Gotkas H.D., Foster H.A., Godwin L.S., Harvey A., Kill I.R., Knight M., Mehta I.S., Ahmed M.H. (2014). The Non-random Repositioning of Whole Chromosomes and Individual Gene Loci in Interphase Nuclei and Its Relevance in Disease, Infection, Aging, and Cancer. Adv. Exp. Med. Biol..

[3] Boyle S., Gilchrist S., Bridger J.M., Mahy N.L., Ellis J.A., Bickmore W.A. (2001). The spatial organization of human chromosomes within the nuclei of normal and emerin-mutant cells. Hum. Mol. Genet..

[4] Bolzer A., Kreth G., Solovei I., Koehler D., Saracoglu K., Fauth C., Müller S., Eils R., Cremer C., Speicher M.R. (2005). Three dimensional maps of all chromosomes in human male fibroblast nuclei and prometaphase rosettes. PLoS Biol..

[5] Saccone S., Federico C., Bernardi G. (2002). Localization of the gene-richest and the gene poorest isochores in the interphase nuclei of mammals and birds. Gene.

[6] Federico C., Cantarella C.D., Di Mare P., Tosi S., Saccone S. (2008). The radial arrangement of the human chromosome 7 in the lymphocyte cell nucleus is associated with chromosomal band gene density. Chromosoma.

[7] Meaburn K.J., Newbold R.F., Bridger J.M. (2008). Positioning of human chromosomes in murine cell hybrids according to synteny. Chromosoma.

[8] Mayer R., Brero A., von Hase J., Schroeder T., Cremer T., Dietzel S. (2005). Common themes and cell type specific variations of higher order chromatin arrangements in the mouse. BMC Cell Biol..

[9] Tanabe H., Mùller S., Neusser M., von Hase J., Calcagno E., Cremer M., Solovei S., Cremer C., Cremer T. (2002). Evolutionary conservation of chromosome territory arrangements in cell nuclei from higher primates. Proc. Natl. Acad. Sci. USA.

[10] Federico C., Pappalardo A.M., Ferrito V., Tosi S., Saccone S. (2017). Genomic properties of chromosomal bands are linked to evolutionary rearrangements and new centromere formation in primates. Chromosome Res..

[11] Kociucka B., Sosnowski J., Kubiak A., Nowak A., Pawlak P., Szczerbal I. (2013). Three-Dimensional Positioning of B Chromosomes in Fibroblast Nuclei of the Red Fox and the Chinese Raccoon Dog. Cytogenet. Genome Res..

[12] Foster H.A., Griffin D.K., Bridger J.M. (2012). Interphase chromosome positioning in in vitro porcine cells and ex vivo porcine tissues. BMC Cell Biol..

[13] Federico C., Saccone S., Andreozzi L., Motta S., Russo V., Carels N., Bernardi G. (2004). The pig genome: Compositional analysis and identification of the gene-richest regions in chromosomes and nuclei. Gene.

[14] Habermann F.A., Cremer M., Walter J., Kreth G., von Hase J., Bauer K., Wienberg J., Cremer C., Cremer T., Solovei I. (2001). Arrangements of macro- and microchromosomes in chicken cells. Chromosome Res..

[15] Federico C., Cantarella C.D., Scavo C., Saccone S., Bed’Hom B., Bernardi G. (2005). Avian genomes: Different karyotypes but a similar distribution of the GC-richest chromosome regions at interphase. Chromosome Res..

[16] Federico C., Scavo C., Cantarella C.D., Motta S., Saccone S., Bernardi G. (2006). Gene-rich and gene-poor chromosomal regions have different locations in the interphase nuclei of cold-blooded vertebrates. Chromosoma.

[17] Croft J., Bridger J.M., Boyle S., Perry P., Teague P., Bickmore W.A. (1999). Differences in the localization and morphology of chromosomes in the human nucleus. J. Cell Biol..

[18] Foster H.A., Abeydeera L.R., Griffin D.K., Bridger J.M. (2005). Non-random chromosome positioning in mammalian sperm nuclei, with migration of the sex chromosomes during late spermatogenesis. J. Cell Sci..

[19] Dekker J., Rippe K., Dekker M., Kleckner N. (2002). Capturing Chromosome Conformation. Science.

[20] Guelen L., Pagie L., Brasset E., Meuleman W., Faza M.B., Talhout W., Eussen B.H., De Klein A., Wessels L., De Laat W. (2008). Domain organization of human chromosomes revealed by mapping of nuclear lamina interactions. Nature.

[21] Bourne G., Moir C., Bikkul U., Ahmed Hassan M., Kill I.R., Eskiw C.H., Tosi S., Yurov Y., Vorsanova S., Iourov I. (2013). Interphase chromosome behavior in normal and diseased cells. Human Interphase Chromosomes.

[22] Xing Y., Johnson C.V., Moen P.T., McNeil J.A., Lawrence J. (1995). Nonrandom gene organization: Structural arrangements of specific pre-mRNA transcription and splicing with SC-35 domains. J. Cell Biol..

[23] Mahy N.L., Perry P.E., Gilchrist S., Baldock R.A., Bickmore W.A. (2002). Spatial organization of active and inactive genes and noncoding DNA within chromosome territories. J. Cell Biol..

[24] D’Antoni S., Mattina T., Di Mare P., Federico C., Motta S., Saccone S. (2004). Altered replication timing of the HIRA/Tuple1 locus in the DiGeorge and Velocardiofacial syndromes. Gene.

[25] Ballabio E., Cantarella C.D., Federico C., Di Mare P., Hall G., Harbott J., Hughes J., Saccone S., Tosi S. (2009). Ectopic expression of the HLXB9 gene is associated with an altered nuclear position in t(7;12) leukaemias. Leukemia.

[26] Leotta C.G., Federico C., Brundo M.V., Tosi S., Saccone S. (2014). HLXB9 gene expression, and nuclear location during in vitro neuronal differentiation in the SK-N-BE neuroblastoma cell line. PLoS ONE.

[27] Gulino G.M., Bruno F., Sturiale V., Brancato D., Ragusa D., Tosi S., Saccone S., Federico C. (2021). From FISH to Hi-C: The chromatin architecture of the chromosomal region 7q36.3, frequently rearranged in leukemic cells, is evolutionary conserved. Int. J. Mol. Sci..

[28] Szczerbal I., Foster H.A., Bridger J.M. (2009). The spatial repositioning of adipogenesis genes is correlated with their expression status in a porcine mesenchymal stem cell adipogenesis model system. Chromosoma.

[29] Szczerbal I., Bridger J.M. (2010). Association of adipogenic genes with SC-35 domains during porcine adipogenesis. Chromosome Res..

[30] Arican-Goktas H.D., Ittiprasert W., Bridger J.M., Knight M. (2014). Differential Spatial Repositioning of Activated Genes in Biomphalaria glabrata Snails Infected with Schistosoma mansoni. PLoS Negl. Trop. Dis..

[31] Cremer T., Cremer C. (2001). Chromosome territories, nuclear architecture and gene regulation in mammalian cells. Nat. Rev. Genet..

[32] Malhas A., Lee C.F., Sanders R., Saunders N.J., Vaux D.J. (2007). Defects in lamin B1 expression or processing affect interphase chromosome position and gene expression. J. Cell Biol..

[33] Saccone S., De Sario A., Della Valle G., Bernardi G. (1992). The highest gene concentrations in the human genome are in telomeric bands of metaphase chromosomes. Proc. Natl. Acad. Sci. USA.

[34] Saccone S., De Sario A., Wiegant J., Raap A.K., Della Valle G., Bernardi G. (1993). Correlations between isochores and chromosomal bands in the human genome. Proc. Natl. Acad. Sci. USA.

[35] Saccone S., Federico C., Solovei I., Croquette M.F., Della Valle G., Bernardi G. (1999). Identification of the gene-richest bands in human prometaphase chromosomes. Chromosome Res..

[36] Federico C., Andreozzi L., Saccone S., Bernardi G. (2000). Gene density in the Giemsa bands of human chromosomes. Chromosome Res..

[37] Bickmore W.A., van Steensel B. (2013). Genome Architecture: Domain Organization of Interphase Chromosomes. Cell.

[38] Simonis M., Klous P., Splinter E., Moshkin Y., Willemsen R., de Wit E., van Steensel B., de Laat W. (2006). Nuclear organization of active and inactive chromatin domains uncovered by chromosome conformation capture-on-chip (4C). Nat. Genet..

[39] Dostie J., Richmond T.A., Arnaout R.A., Selzer R.R., Lee W.L., Honan T.A., Rubio E.D., Krumm A., Lamb J., Nusbaum C. (2006). Chromosome Conformation Capture Carbon Copy (5C): A massively parallel solution for mapping interactions between genomic elements. Genome Res..

[40] Lieberman-Aiden E., van Berkum N.L., Williams L., Imakaev M., Ragoczy T., Telling A., Amit I., Lajoie B.R., Sabo P.J., Dorschner M.O. (2009). Comprehensive Mapping of Long-Range Interactions Reveals Folding Principles of the Human Genome. Science.

[41] Belton J.-M., McCord R.P., Gibcus J.H., Naumova N., Zhan Y., Dekker J. (2012). Hi-C: A comprehensive technique to capture the conformation of genomes. Methods.

[42] Shavit Y., Lio’ P. (2013). CytoHiC: A cytoscape plugin for visual comparison of Hi-C networks. Bioinformatics.

[43] Botta M., Haider S., Leung I.X.Y., Lio’ P., Mozziconacci J. (2010). Intra- and inter-chromosomal interactions correlate with CTCF binding genome wide. Mol. Syst. Biol..

[44] Jabbari K., Bernardi G. (2017). An Isochore Framework Underlies Chromatin Architecture. PLoS ONE.

[45] Anania C., Lupianez D.G. (2020). Order and disorder: Abnormal 3D chromatin organization in human disease. Brief. Funct. Genom..

[46] Qiu Y., Huang S. (2020). CTCF mediated genome organization and leukemogenesis. Leukemia.

[47] Wang S., Su J.-H., Beliveau B.J., Bintu B., Moffitt J.R., Wu C., Zhuang X. (2016). Spatial organization of chromatin domains and compartments in single chromosomes. Science.

[48] Stevens T.J., Lando D., Basu S., Atkinson L.P., Cao Y., Lee S.F., Leeb M., Wohlfahrt K.J., Boucher W., O’Shaughnessy-Kirwan A. (2017). 3D structures of individual mammalian genomes studied by single-cell Hi-C. Nature.

[49] Bernardi G. (2015). Chromosome Architecture and Genome Organization. PLoS ONE.

[50] Kurz A., Lampel S., Nickolenko J.E., Bradl J., Benner A., Zirbel R.M., Cremer T., Lichter P. (1996). Active and inactive genes localize preferentially in the periphery of chromosome territories. J. Cell Biol..

[51] Dietzel S., Schiebel K., Little G., Edelmann P., Rappold G.A., Eils R., Cremer C., Cremer T. (1999). The 3D Positioning of *ANT2* and *ANT3* Genes within Female X Chromosome Territories Correlates with Gene Activity. Exp. Cell Res..

[52] Scheuermann M.O., Tajbakhsh J., Kurz A., Saracoglu K., Eils R., Lichter P. (2004). Topology of genes and nontranscribed sequences in human interphase nuclei. Exp. Cell Res..

[53] Galiovà G., Bàrtovà E., Kozubek S. (2004). Nuclear topography of beta-like globin gene cluster in IL-3-stimulated human leukemic K-562 cells. Blood Cells Mol. Dis..

[54] Tajbakhsh J., Luz H., Bornfleth H., Lampel S., Cremer C., Lichter P. (2000). Spatial Distribution of GC- and AT-Rich DNA Sequences within Human Chromosome Territories. Exp. Cell Res..

[55] Torabi K., Wangsa D., Ponsa I., Brown M., Bosch A., Vila-Casadesùs M., Karpova T.S., Calvo M., Castells A., Miró R. (2017). Transcription-dependent radial distribution of TCF7L2 regulated genes in chromosome territories. Chromosoma.

[56] Peng A.Y.T., Kolhe J.A., Behrens L.D., Freeman B.C. (2021). Genome organization: Tag it, move it, place it. Curr. Op. Cell Biol..

[57] Gandhi M.S., Stringer J.R., Nikiforova M.N., Medvedovic M., Nikiforov Y.E. (2009). Gene position within chromosome territories correlates with their involvement in distinct rearrangement types in thyroid cancer cells. Genes Chromosomes Cancer.

[58] Verschure P.J., van der Kraan I., Enserink J.M., Moné M.J., Manders E.M.M., van Driel R. (2002). Large-scale Chromatin Organization and the Localization of Proteins Involved in Gene Expression in Human Cells. J. Histochem. Cytochem..

[59] Branco M.R., Pombo A. (2006). Intermingling of Chromosome Territories in Interphase Suggests Role in Translocations and Transcription-Dependent Associations. PLoS Biol..

[60] Volpi E.V., Chevret E., Jones T., Vatcheva R., Williamson J., Beck S., Campbell R.D., Goldsworthy M., Powis S.H., Ragoussis J. (2000). Large-scale chromatin organization of the major histocompatibility complex and other regions of human chromosome 6 and its response to interferon in interphase nuclei. J. Cell Sci..

[61] Williams R.R.E., Broad S., Sheer D., Ragoussis J. (2002). Subchromosomal Positioning of the Epidermal Differentiation Complex (EDC) in Keratinocyte and Lymphoblast Interphase Nuclei. Exp. Cell Res..

[62] Khanna N., Hu Y., Belmont A.S. (2014). HSP70 Transgene Directed Motion to Nuclear Speckles Facilitates Heat Shock Activation. Curr. Biol..

[63] Lahbib-Mansais Y., Barasc H., Marti-Marimon M., Mompart F., Iannuccelli E., Robelin D., Riquet J., Yerle-Bouissou M. (2016). Expressed alleles of imprinted IGF2, DLK1 and MEG3 colocalize in 3Dpreserved nuclei of porcine fetal cells. BMC Cell Biol..

[64] Bell S.P., Dutta A. (2002). DNA Replication in Eukaryotic Cells. Annu. Rev. Biochem..

[65] Rivera-Mulia J.C., Gilbert D.M. (2016). Replication timing and transcriptional control: Beyond cause and effect—Part III. Curr Opin. Cell Biol..

[66] Chagin V.O., Casas-Delucchi C.S., Reinhart M., Schermelleh L., Markaki Y., Maiser A., Bolius J.J., Bensimon A., Fillies M., Domaing P. (2016). 4D Visualization of replication foci in mammalian cells corresponding to individual replicons. Nat. Commun..

[67] Meister P., Taddei A., Gasser S.M. (2006). In and out of the Replication Factory. Cell.

[68] Ryba T., Hiratani I., Lu J., Itoh M., Kulik M., Zhang J., Schulz T.C., Robins A.J., Dalton S., Gilbert D.M. (2010). Evolutionarily conserved replication timing profiles predict long-range chromatin interactions and distinguish closely related cell types. Genome Res..

[69] Zink D. (2006). The temporal program of DNA replication: New insights into old questions. Chromosoma.

[70] Drouin R., Lemieux N., Richer C.-L. (1991). Chromosome condensation from prophase to late metaphase: Relationship to chromosome bands and their replication time. Cytogenet. Genome Res..

[71] Rhind N., Gilbert D.M. (2013). DNA Replication Timing. Cold Spring Harb. Perspect. Biol..

[72] Woodfine K., Fiegler H., Beare D.M., Collins J.E., McCann O.T., Young B.D., Debernardi S., Mott R., Dunham I., Carter N.P. (2004). Replication timing of the human genome. Hum. Mol. Genet..

[73] Dutrillaux B., Couturier J., Richer C.-L., Viegas-Pequinot E. (1976). Sequence of DNA replication in 277 R- and Q-bands of human chromosomes using a BrdU treatment. Chromosoma.

[74] Federico C., Saccone S., Bernardi G. (1998). The gene-richest bands of human chromosomes replicate at the onset of the S-phase. Cytogenet. Genome Res..

[75] Grasser F., Neusser M., Fiegler H., Thormeyer T., Cremer M., Carter N.P., Cremer T., Müller S. (2008). Replication-timing-correlated spatial chromatin arrangements in cancer and in primate interphase nuclei. J. Cell Sci..

[76] Ferreira J., Paolella G., Ramos C., Lamond A.L. (1997). Spatial organization of large-scale chromatin domains in the nucleus: A magnified view of single chromosome territories. Cell Biol..

[77] Sadoni N., Langer S., Fauth C., Bernardi G., Cremer T., Turner B.M., Zink D. (1999). Nuclear organization of mammalian genomes: Polar chromosome territories build up functionally distinct higher order compartments. J. Cell Biol..

[78] Donley N., Thayer M.J. (2013). DNA replication timing, genome stability and cancer. Semin. Cancer Biol..

[79] Boggs B.A., Chinault A.C. (1994). Analysis of replication timing properties of human Xchromosomal loci by fluorescence in situ hybridization. Proc. Natl. Acad. Sci. USA.

[80] Chess A., Simon I., Cedar H., Axel R. (1994). Allelic inactivation regulates olfactory receptor gene expression. Cell.

[81] Knoll J.H.M., Cheng S.-D., Lalande M. (1994). Allele specificity of DNA replication timing in the Angelman/Prader-Willi syndrome imprinted chromosomal region. Nat. Genet..

[82] Simon I., Tenzen T., Reubinoff B.E., Hillman D., McCarrey J.R., Cedar H. (1999). Asynchronous replication of imprinted genes is established in the gametes and maintained during development. Nature.

[83] Reish O., Orlovski A., Mashevitz M., Sher C., Libman V., Rosenblat M., Avivi L. (2003). Modified allelic replication in lymphocytes of patients with neurofibromatosis type 1. Cancer Genet. Cytogenet..

[84] Laish I., Mannasse-Green B., Hadary R., Konikoff F.M., Amiel A., Kitay-Cohen Y. (2016). Aneuploidy and asynchronous replication in non-alcholic fatty liver disease and cryptogenic cirrhosis. Gene.

[85] Amiel A., Elis A., Blumenthal D., Gaber E., Fejgin M.D., Dubinsky R., Lishner M. (2001). Modified order of allelic replication in lymphoma patients at different disease stages. Cancer Genet. Cytogenet..

[86] Korenstein-Ilan A., Amiel A., Lalezari S., Lishner M., Avivi L. (2002). Allele-specific replication associated with aneuploidy in blood cells of patients with hematologic malignancies. Cancer Genet. Cytogenet..

[87] Dotan Z.A., Dotan A., Ramon J., Avivi L. (2008). Aberrant allele-specific replication, independent of parental origin, in blood cells of cancer patients. BMC Cancer.

[88] Grinberg-Rashi H., Cytron S., Gelman-Kohan Z., Litmanovitch T., Avivi L. (2010). Replication timing aberrations and aneuploidy in peripheral blood lymphocytes of breast cancer patients. Neoplasia.

[89] Nagler R.H., Gray S.W., Romantan A., Kelly B.J., DeMichele A., Armstrong K., Schwartz J.S., Hornik R.C. (2010). Differences in information seeking among breast, prostate, and colorectal cancer patients: Results from a population-based survey. Patient Educ. Couns..

[90] Ryba T., Battaglia D., Chang B.H., Shirley J.W., Buckley Q., Pope B.D., Devidas M., Druker B.J., Gilbert D.M. (2012). Abnormal developmental control of replication-timing domains in pediatric acute lymphoblastic leukemia. Genome Res..

[91] Fritz A., Sinha S., Marella N., Berezney R. (2013). Alterations in replication timing of cancer related genes in malignant human breast cancer cells. J. Cell Biochem..

[92] Wang H., Han M., Qi L.S. (2021). Engineering 3D genome organization. Nat. Rev. Genet..

[93] Harewood L., Fraser P. (2014). The impact of chromosomal rearrangements on regulation of gene expression. Hum. Mol. Genet..

[94] Gheldof N., Witwicki R., Migliavacca E., Leleu M., Didelot G., Harewood L., Rougemont J., Reymond A. (2013). Structural variation-associated expression changes are paralleled by chromatin architecture modifications. PLoS ONE.

[95] Ballabio A., Gieselmann V. (2009). Lysosomal disorders: From storage to cellular damage. Biochim. Biophys. Acta.

[96] Harewood L., Schutz F., Boyle S., Perry P., Delorenzi M., Bickmore W.A., Reymond A. (2010). The effect of translocation-induced nuclear reorganization on gene expression. Genome Res..

[97] Federico C., Owoka T., Ragusa D., Sturiale V., Caponnetto D., Leotta C.G., Bruno F., Foster H.A., Rigamonti S., Giudici G. (2019). Deletions of chromosome 7q affect nuclear organization and *HLXB9* gene expression in hematological disorders. Cancers.

[98] Taslerovà R., Kozubek S., Lukàsovà E., Jirsovà P., Bàrtovà E., Kozubek M. (2003). Arrangement of chromosome 11 and 22 territories, EWSR1 and FLI1 genes, and other genetic elements of these chromosomes in human lymphocytes and Ewing sarcoma cells. Hum. Genet..

[99] Lukàsovà E., Kozubek S., Kozubek M., Kjeronskà J., Ryznar L., Horàkovà J., Horneck G. (1997). Localisation and distance between ABL and BCR genes in interphase nuclei of bone marrow cells of control donors and patients with chronic myeloid leukaemia. Hum. Genet..

[100] Murmann A.E., Gao J., Encinosa M., Gautier M., Peter M.E., Eils R., Lichter P., Rowley J.D. (2005). Local gene density predicts the spatial position of genetic loci in the interphase nucleus. Exp. Cell Res..

[101] Meaburn K.J., Misteli T. (2008). Locus-specific and activity-independent gene repositioning during early tumorigenesis. J. Cell Biol..

[102] Morey C., Da Silva N.R., Kmita M., Duboule D., Bickmore W.A. (2008). Ectopic nuclear reorganization driven by a Hoxb1 transgene transposed into Hoxd. J. Cell Sci..

[103] Federico C., Leotta C.G., Bruno F., Longo A.M., Owoka T., Tosi S., Saccone S. (2017). Nuclear repositioning of the non-translocated HLXB9 allele in the leukaemia cell line GDM-1 harbouring a t(6;7)(q23;q36). Cytogenet. Genome Res..

[104] Roix J.J., McQueen P.G., Munson P.J., Parada L.A., Misteli T. (2003). Spatial proximity of translocation-prone gene loci in human lymphomas. Nat. Genet..

